# When do anorexic patients perceive their body as too fat? Aggravating and ameliorating factors

**DOI:** 10.1371/journal.pone.0212612

**Published:** 2019-02-22

**Authors:** Miguel Kazén, Nicola Baumann, Janne F. Twenhöfel, Julius Kuhl

**Affiliations:** 1 Institute of Psychology, University of Osnabrück, Osnabrück, Germany; 2 Department of Psychology, University of Trier, Trier, Germany; University of Rome, ITALY

## Abstract

**Objective:**

Our study investigated body image representations in female patients with anorexia nervosa and healthy controls using a size estimation with pictures of their own body. We also explored a method to reduce body image distortions through right hemispheric activation.

**Method:**

Pictures of participants' own bodies were shown on the left or right visual fields for 130 ms after presentation of neutral, positive, or negative word primes, which could be self-relevant or not, with the task of classifying the picture as “thinner than”, “equal to”, or “fatter than” one’s own body. Subsequently, activation of the left- or right hemispheric through right- or left-hand muscle contractions for 3 min., respectively. Finally, participants completed the size estimation task again.

**Results:**

The distorted “fatter than” body image was found only in patients and only when a picture of their own body appeared on the right visual field (left hemisphere) and was preceded by negative self-relevant words. This distorted perception of the patients' body image was reduced after left-hand muscle contractions (right hemispheric activation).

**Discussion:**

To reduce body image distortions it is advisable to find methods that help anorexia nervosa patients to increase their self-esteem. The body image distortions were ameliorated after right hemispheric activation. A related method to prevent distorted body-image representations in these patients may be Eye Movement Desensitization and Reprocessing (EMDR) therapy.

## Introduction

The body image plays an essential role in the development of attitudes towards eating. The term body image refers to the representation of the body's size, shape, and weight as well as our own feelings towards it [1]. Negative thoughts and dysfunctional assumptions related to body size, shape, and weight are key etiological and maintenance factors in eating disorders, such as anorexia nervosa [2], [3].

How are body images represented in anorexia nervosa patients? There is evidence of hemispheric differences, suggesting a preponderant role to the left hemisphere in body image distortions. Smeets and Kosslyn [4] showed real and distorted (thinner or fatter) pictures of participants' own bodies and of a famous person’s body on the left or right visual fields very briefly (130 ms) to patients with anorexia nervosa and to healthy controls. Particpants' task was to indicate whether the picture was thinner than, equal to, or fatter than their own actual body size. Healthy participants showed no hemispheric differences and made accurate size judgments, of both their own and a famous person’s body. Anorexic patients, in contrast, judged a higher proportion of fatter distortions as being equal to their own body size and this effect was stronger and significant for left-hemispheric (right visual field) presentations. Their judgments of a famous person's body, however, were comparable to those of healthy controls. In other words, the body image distortions of anorexic patients were specific to their own bodies and were related to activation of the left hemisphere.

Left-hemispheric activation has been associated with distorted information about the self in persons unable to reduce negative affect under stress (state oriented). This has been demonstrated in studies dealing with self-infiltration, which is the tendency to falsely ascribe norms or tasks originated by others to oneself [5]. Self-infiltration is often accompanied by feelings of shame, pressure, compulsion, and alienation [6], [7]. Stronger self-infiltration has been observed after right-hand (left hemisphere) compared to left-hand (right hemisphere) muscle contractions [8]. This finding connects left hemispheric activation with introjection, which is one form of norm internalization [9], [10].

Personality systems interactions (PSI) theory [9], [11] (see also [12]), proposes two main forms of self-government: Self-control and self-regulation. Self-control is an autocratic form of executive functioning in which an individual's main goal has upmost priority, and any other needs and goals are suppressed. It is associated with intention memory and left-hemispheric activation. Intention memory has weak connections with bodily processes and represents idealized images of one’s self ("introjects"). Self-regulation is a more democratic form of executive functioning in which an individual's main goal is pursued while attempting to integrate all of her/his needs and goals instead of ignoring them. It is associated with extension memory and right-hemispheric activation. Extension memory has strong connections with bodily processes and with veridical representations of the self (see [9], [11], [13]).

Anorexic patients are extremely self-disciplined and refrain from eating even if they are hungry. This indicates that they frequently use the self-control mode to attain their goal of weight reduction, which is effortful and could lead to ego-depletion [14], [15]. Based on PSI theory, we propose that anorexic patients have consciously or unconsciously introjected a false body image of themselves that bears little correspondence with their actual body shape. If that is true we can expect that anorexic patients activate the left hemisphere to represent schematic and unrealistic representations of their ideal body size and shape [4]. Consistent with this idea, anorexic patients with strong right-handedness, indicating dominance of the left hemisphere, show a larger discrepancy between actual and perceived body mass index (BMI; [16]). That is, these patients perceive themselves as fatter (larger MBI) than they actually are.

Furthermore, areas of the right hemisphere such as the right lateral temporal-occipital cortex, are activated during body-image perception, as indicated by fMRI studies [17]. It has been suggested that the right hemisphere maintains accurate body representations, which are lost after specific lesions. This is in line with PSI theory and has been observed in reports of patients with “Anosognosia” (deficit in self-awareness), who have right parietal lobe lesions. These patients are paralyzed on one side of their body but deny their paralysis [18]. However, if the right hemisphere is temporarily stimulated these patients acknowledge their paralysis during stimulation, and denial of paralysis returns when the stimulation ceases [19]. In healthy individuals there is evidence that increments in the activity and size of the right insular cortex are related to interoceptive awareness [20], which is also related to perception of one’s own body.

Consistent with the notion that anorexic patients' deficit in body image perception is related to an under-activation of the right hemisphere, these patients show deficits in tests of right parietal lobe functioning [21] and abnormal event-related activity in central right-parietal areas [22], [23]. Moreover, anorexic patients show decreased attention when looking at pictures of their own bodies, and this diminished attention is related to under-activation of the right inferior parietal lobe [24]. In sum, there seems to be a right hemispheric deficit in anorexic patients in perceiving and evaluating actual pictures of their own body.

The aim of this study is to investigate body-image representations of anorexic patients. Based on the findings of Smeets and Kosslyn ([4]) we expected anorexic patients to perceive pictures of their own body as fatter than they actually are when they are presented in the right visual field (left hemisphere) but not when they are presented in the left visual field (right hemisphere). The present study investigates two issues.

The first issue addresses whether body image distortions occur independently from individual's emotional states or under specific emotional states. A distorted body image in anorexia nervosa is marked by dysfunctional thoughts and negative feelings towards one’s own body [25], [26], which are also related to low levels of self-esteem and high levels of perfectionism [27]. In addition, it has been speculated that anorexia nervosa should be considered a disorder of the self [28], [29], which is rooted in attachment insecurity during development [30]. To examine this issue and take all of the aformentioned considerations into account, we induced emotional states by presenting positive or negative word primes that were related or not related to the self. After each prime, a picture of the participant’s body was briefly presented in the left or right visual field. Participant's task was to classify the picture as thinner than, equal to, or fatter than their own body. As a further control we included emotionally neutral primes. Our hypothesis is that body image distortions should occur with *negative self-related* primes followed by a picture of the body on the right visual field →left hemisphere (cf. Smeets & Kosslyn). Positive and neutral primes are not expected to boost body-image distortions.

The second issue that we explore, if confirmed, could be of practical significance. We peopose that body image distortions of anorexic patients occur because patients compare their actual body size with idealized unrealistic schemas of their own body (introjects), which are represented in their left hemisphere. Consequently, we can expect that activation of patients' right hemisphere through unilateral muscle contractions of the left hand would reduce body image distortions. No mitigating effect on body image distortions is expected after muscle contractions of the right hand. There are experimental ([8], [31], [32], [33]) and EEG studies ([34], [35]) showing that unilateral muscle contractions activate the contralateral hemisphere. It has been found that activation of the right hemisphere through unilateral muscle contractions reduces self-infiltration [8], enhances creative thinking [32], increases implicit self-esteem [36], and prevents chocking under pressure in athletes, presumably by suppressing conscious control of the left hemisphere [31]. We therefore included activation of the left or right hemisphere as within-participant variable in the design. In particular, participants carried out unilateral muscle contractions for 3 min with the left hand and with the right hand (counterbalanced across participants) *after* the main task was completed and before they repeated the set of experimental trials in each condition.

In sum, our first hypothesis is that body-image distortions of anorexic patients should occur with *negative self-related* primes followed by a picture of their own body presented on the right visual field (left hemisphere) but not on the left visual field (right hemisphere). Our second hypothesis is that activation of the right hemisphere through unilateral muscle contractions of the left hand should reduce body-image distortions.

## Method

### Participants

A total of 42 participants voluntarily participated in the study. Twenty of them were female patients diagnosed with anorexia nervosa. All patients were undergoing cognitive-behavioral therapy and received medication for depression. Mean age was 26.5 (*SD* = 9.88) years. The patients were formally diagnosed by qualified psychotherapists. According to the ICD-10-CM, twelve of the anorexic patients fulfilled the criteria for anorexia nervosa disorder (F50.0) and eight of the patients fulfilled the criteria for anorexia nervosa binge eating/purging type (F50.02). Their average BMI was 18.9 (*SD* = 1.81), with a range from 15.8 to 22.5 (the unusual highest BMI score was from an anorexic patient with binge eating). There was also a group of 22 female controls without a history of eating disorders. They had a mean age of 21.8 (*SD* = 2.04) years. Their average BMI was 21.1 (*SD* = 2.10), with a range from 17.1 to 26.0.

The study was approved by the Ethics Committee of the University of Osnabrück, Germany. The patients were recruited from a clinic in a nearby German city and were thoroughly informed about the study and use of the data. Paticipation in the study was anonymous and voluntary (participants could quit any time they wanted). They signed a consent form before participating and all participants completed the study. Some patients were tested in the clinic and others in the university laboratory. The participants in the control group were mainly university students. They were also informed about the nature of the study in advance and signed a consent form before participating.

### Self-report measures

#### Volitional components inventory

(VCI; [37]) was applied to assess participants’ self-rated competence in self-regulation and stress management ([38]). The short-version consists of 13 scales with 4 items each. Responses are given on a 4-point Likert scale [*not at all* (0), *sometimes* (1), *frequently* (2), *or constantly* (3)"]. In the present study, the following scales were used: Self-determination (α = 0.76), “*I feel that most of the time I really want to do the things I do*.” Self-motivation (α = 0.82), “I *am capable of finding the pleasant aspects of an initially unpleasant activity*,” and Self-access (α = 0.78), “*When I’m under pressure*, *I lose access to my feelings*.” (reversed). In addition, two scales measuring stress were applied. Threats (α = 0.83), “*My current life circumstances are very tough*,” and Demands (α = 0.82), “*There have been many changes in my life*, *which I need to cope with*.” The VCI has been extensively validated (see e.g., [38], [39], [40]).

#### Action control scale

The Action Control Scale is a personality measure of action control (ACS; [41]). We applied the failure and the decision related scales (each with a Cronbach’s α = 0.78). An example item from the failure or threat scale (AOF): "*When I am told that my work has been completely unsatisfactory*: *(a) I don’t let it bother me for too long*, *or (b) I feel paralyzed*." The option "*a*" reflects the action-oriented and option "*b*" the state-oriented alternative. The scale ranges from 0–12 with higher scores indicating action orientation (*disengagement*) and lower scores state orientation (*preoccupation*). An example of the decision and initiative scale (AOD): "*When I have to carry out an important but unpleasant task*: *(a) I do it and get it over with*, *or (b) It can take a while before I can bring myself to do it*." The option "(*a*)" illustrates the action-oriented and "(*b*)" the state-oriented alternative. The scale ranges from 0–12 with higher scores indicating action orientation (*initiative or decisiveness*) and lower scores state orientation (*hesitation*). This ACS scale has a well-established construct validity in research and applied settings ([42]; see also [43], [44]).

#### Thought control questionnaire-eating

(TCQ-E; Kazén & Twenhöfel, [45]**).** To assess the ability to control thoughts an instrument which took as model the thought control questionnaire (TCQ) of Wells and Davies [46] (German translation by Fehm and Hoyer, [47]) was applied. The TCQ-E has 6 scales with 3 items each. Responses are given on a 4-point Likert scale [*not at all* (0), *sometimes* (1), *frequently* (2), *or constantly* (3)"]. The first two scales measure general thought control: (1) Negative affect (Cronbach’s α = 0.72) *"When I want to suppress a particular thought and I fail*, *I punish myself*.*”* and (2) Positive affect (α = 0.60) *"I love to imagine beautiful pictures or stories with my thoughts*.*”* There are four scales related specifically to thought control about eating. (3) Low impulse control (α = 0.89) “*When I see something delicious to eat*, *I am not able to restrain myself*.” (4) Passive coping (α = 0.59) “*I allow myself thoughts about eating*, *because I know that they will go away*.*”* (5) Distraction (α = 0.70) “*To avoid thinking about eating I keep busy doing other things*.” (6) Negative emotionality (α = 0.81) “*I punish myself when I must all too often think about eating*.”

### Materials

#### Primes

To increase the effectiveness of the primes we let each participant choose her own primes. We presented a list of 246 adjectives in a spreadsheet, half of them more positive and half more negative, and asked participants to classify each adjective according to valence using a 10-point Likert scale: [*very negative* (1), *negative* (3), *neither negative nor positive* (5–6), *positive* (8), *very positive* (10)]. We then presented the adjectives in a second spreadsheet in a different order and asked participants to classify each of them according to self-description using a 10-point Likert scale, “The adjective is characteristic of me: [*not at all* (1–2), *to some extent* (5–6), *completely* (9–10)]. We used these ratings to select 5 idiosyncratic types of items as primes from each of the following sets: *Negative No-Self* (valence: 1–2 and self-description: 1–2), *Negative Self* (valence: 1–2 and self-description: 9–10), *Neutral* (valence: 5–6 and self-description: 5–6), *Positive No-Self* (valence: 9–10 and self-description: 1–2), *Positive Self* (valence: 9–10 and self-description: 9–10).

#### Participant’s pictures

**Fig 1:**
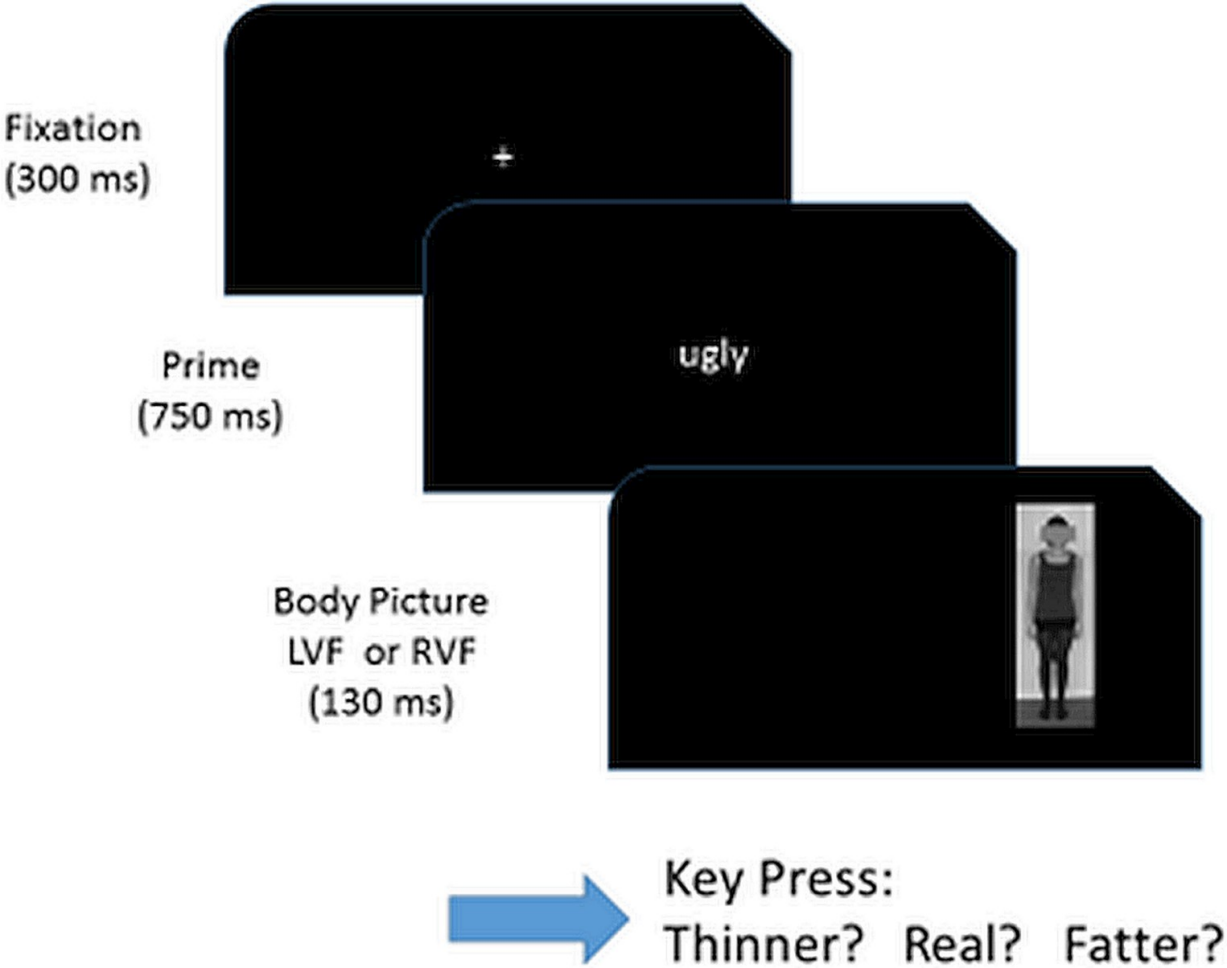
Example of an experimental trial. The primes were generated by the participant herself. In this example, it is a negative self-relevant prime and the picture of her own body was presented on the right visual field (left hemisphere).

Each participant was asked to dress a leotard (gymnastic suit) for a picture of their body to be taken, which was done on a neutral white background. For the experimental trials we put a mask in front of the face of the participant and presented the pictures in black and white (see Fig 1). In addition, those pictures were distorted to make them appear 20% thinner or 20% fatter than they were, using special software. In contrast to the study of Smeets and Kosslyn [4] we did not present pictures with 40% body distortions, either thinner or fatter, because they would be easy to classify as different from one’s own body size. We did also not show a picture of the body of a famous person, only of the participant herself.

### Procedure

The study consisted of two sessions. In the first session, participants completed a series of questionnaires and had their picture taken. In the second session, which took place at least two days after the first session, we prepared the experimental trials using individual primes as described above with the E-Prime program to control experiments [48]. The participants were seated in a noise-reduced room. Participants started each trial themselves by pressing the space bar: There was a fixation cross for 300 ms, followed by a prime for 750 ms, and a participant’s picture for 130 ms (to prevent saccadic eye movements), either in the left- or right visual field. The task was to press one of three possible keys to indicate the perceived body size (J = thinner than my body; K = exact size; L = fatter than my body) as fast as possible. Two participants in the control group were left-handed according to the Edinburgh inventory of handedness [49]. For them, we assigned the keys located on the left side of the keyboard for their responses (A = thinner than my body; S = exact size; D = fatter than my body).

There were a total of 180 trials: 30 primes X 3 body sizes X 2 presentations. After completion of this initial measure, participants were randomly assigned to either the left- or right-hand ball squeezing condition. In each case, after squeezing the ball for 3 min. the participant completed the 180 trials again. That is, each participant carried out the set of experimental trials 3 times (initially, after right-hand/left hemispheric activation, and after left-hand/right hemispheric activation). At the end of the experiment the participant was thanked for her participation and fully debriefed about the nature of the study.

## Results

The main dependent variable was accuracy of judgments of pictures showing the *real body size* of the participant (see Smeets & Kosslyn [4]). Although we have a clear a-priori hypothesis dealing with negative self-related primes shown on the right-visual field (LH) to anorexic patients, we also examined the overall design to find out whether there were other significant effects. To do that, we carried out a mixed-design ANOVA with the factors Group (anorexic, controls), Prime (Positive No-Self, Positive Self, Neutral, Negative No-Self, and Negative Self), Body Size (thinner, real, fatter), Visual Field (left, right), the last three as within-participant factors. The ANOVA yielded a highly significant 4-way interaction: *F*(8, 320) = 19.06, *p* < .0001, partial *η*^2^ = .33.

We then analyzed accuracy data for each prime type separately using a mixed-design ANOVA with the independent variables: Group (anorexic, controls), Body Size (thinner, real, fatter), and Visual Field (left, right). The last two variables were included as within-participant factors. Three ANOVAs yielded only a main effect of Body Size. Not surprisingly the proportion of correct responses to pictures of the real body size (R) were much more accurate than the incorrect responses to thinner than (T) or fatter than (F) own’s body (*p* < .0001). Positive No-Self: *F*(2, 80) = 152.1, *p* = .0001, partial *η*^2^ = .79 (*M*_T_ = .08, *M*_R_ = .70, *M*_F_ = .12). Positive Self: *F*(2, 80) = 118.4, *p* = .0001, partial *η*^2^ = .75 (*M*_T_ = .09, *M*_R_ = .71, *M*_F_ = .16), and Negative No-Self: *F*(2, 80) = 118.3, *p* = .0001, partial *η*^2^ = .74 (*M*_T_ = .08, *M*_R_ = .69, *M*_F_ = .15). Here there was also a difference between the T and F condition (*p* < .05). Descriptive statistics of all conditions are shown in Table 1 (anorexic patients group) and Table 2 (control group).

**Table 1:** “Equal-to-own-size” responses made by anorexia nervosa patients.

	Thinner (False)		REAL		Fatter (False)	
	LVF (RH)	RVF (LH)	LVF (RH)	RVF (LH)	LVF (RH)	RVF (LH)
Neutral	.03 (.*07*)	.09 (.*11*)	.75 (.*26*)	.76 (.*23*)	.23 (.*21*)	.02 (.*08*)
Positive No-Self	.05 (.*11*)	.13 (.*18*)	.67 (.*26*)	.67 (.*23*)	.14 (.*20*)	.16 (.*23*)
Positive Self	.08 (.*17*)	.12 (.*17*)	.65 (.*30*)	.72 (.*24*)	.21 (.*31*)	.18 (.*29*)
Negative No-Self	.04 (.*09*)	.08 (.*13*)	.68 (.*34*)	.66 (.*27*)	.16 (.*24*)	.21 (.*22*)
**Negative Self**	.12 (.*17*)	.12 (.*17*)	.**68**_**a**_ (.*20*)	**.07**_**a**_ (.*11*)	**.19**_**b**_ (.*25*)	**.68**_**b**_ (.*28*)

Mean proportions (*sd* in parentheses) of “equal-to-own-size” responses to actual body size pictures made by the *anorexia nervosa patients* as a function of prime type, picture presented, and visual field (hemisphere).

*Notes*. Means with the same subscript (_a_ or _b_) differ significantly (*p* < .0001) from each other. Responses to “Real” body size are correct. Responses to “Thinner than” or “Fatter than” own body size are incorrect and indicate distortions in body representations.

**Table 2:** “Equal-to-own-size” responses made by control healthy participants.

	Thinner (False)		REAL		Fatter (False)	
	LVF (RH)	RVF (LH)	LVF (RH)	RVF (LH)	LVF (RH)	RVF (LH)
Neutral	.05 (.*13*)	.11 (.*12*)	.73 (.*25*)	.76 (.*17*)	.17 (.*27*)	.11 (.*21*)
Positive No-Self	.07 (.*17*)	.07 (.*13*)	.72 (.*25*)	.71 (.*21*)	.10 (.*18*)	.08 (.*12*)
Positive Self	.05 (.*09*)	.10 (.*16*)	.75 (.*18*)	.72 (.*26*)	.14 (.*21*)	.11 (.*15*)
Negative No-Self	.07 (.*13*)	.11 (.*16*)	.71 (.*22*)	.71 (.*25*)	.13 (.*19*)	.11 (.*20*)
Negative Self	.04 (.*09*)	.08 (.*15*)	.71 (.*25*)	.68 (.*24*)	.15 (.*21*)	.08 (.*16*)

Mean proportions (*sd* in parentheses) of “equal-to-own-size” responses to actual body size pictures made by the *control healthy participants* as a function of prime type, picture presented, and visual field (hemisphere).

*Notes*. Responses to “Real” body size are correct. Responses to “Thinner than” or “Fatter than” own body size are incorrect and indicate distortions in body representations.

The ANOVA with Neutral primes yielded a main effect of Body Size, *F*(2, 80) = 169.1, *p* = .0001, partial *η*^2^ = .81 and a Body Size x Visual Field interaction: *F*(2, 80) = 12.57, *p* = .001, partial *η*^2^ = .24. Participants in both groups made less „thinner than” errors when the pictures were presented in the LVF (RH) than in the RVF (LH): .04 versus .10 (*p* < .05). On the other hand, they made more „fatter than” errors when the pictures were presented in the LVF (RH) than in the RVF (LH): .20 versus .07 (*p* < .001). There were no differences in the proportions of correct responses in the real size condition: LVF (RH), .74 and RVF (LH), .76.

**Fig 2:**
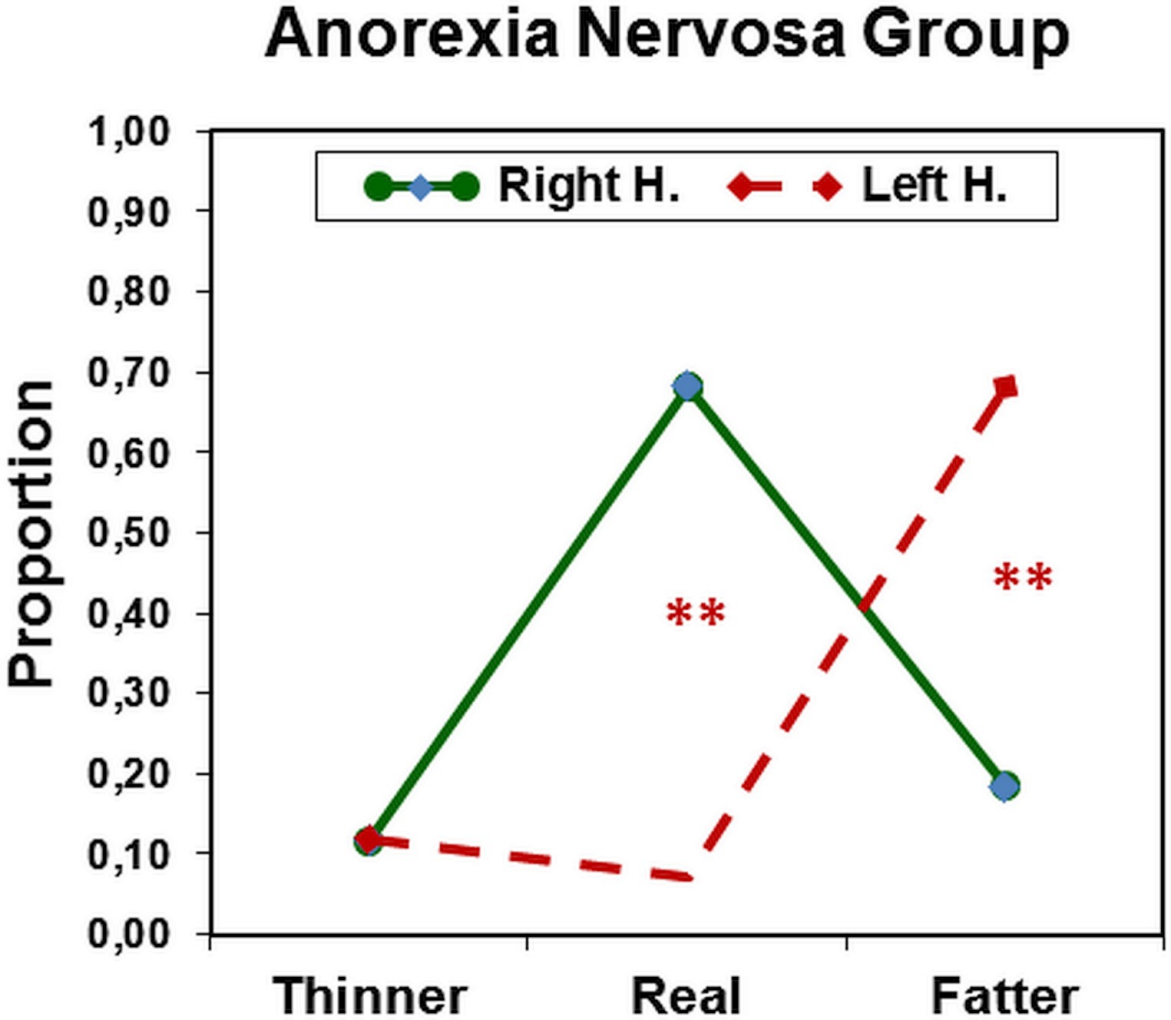
Anorexia nervosa group. Mean proportions of equal-to-own-size judgments as a function of body picture distortion for the left (LH) and right hemisphere (RH), respectively, after negative self-related primes. The contrast between left- and right hemispheres was highly significant both for real (**p < .0001) and fatter (**p < .0001) conditions.

**Fig 3:**
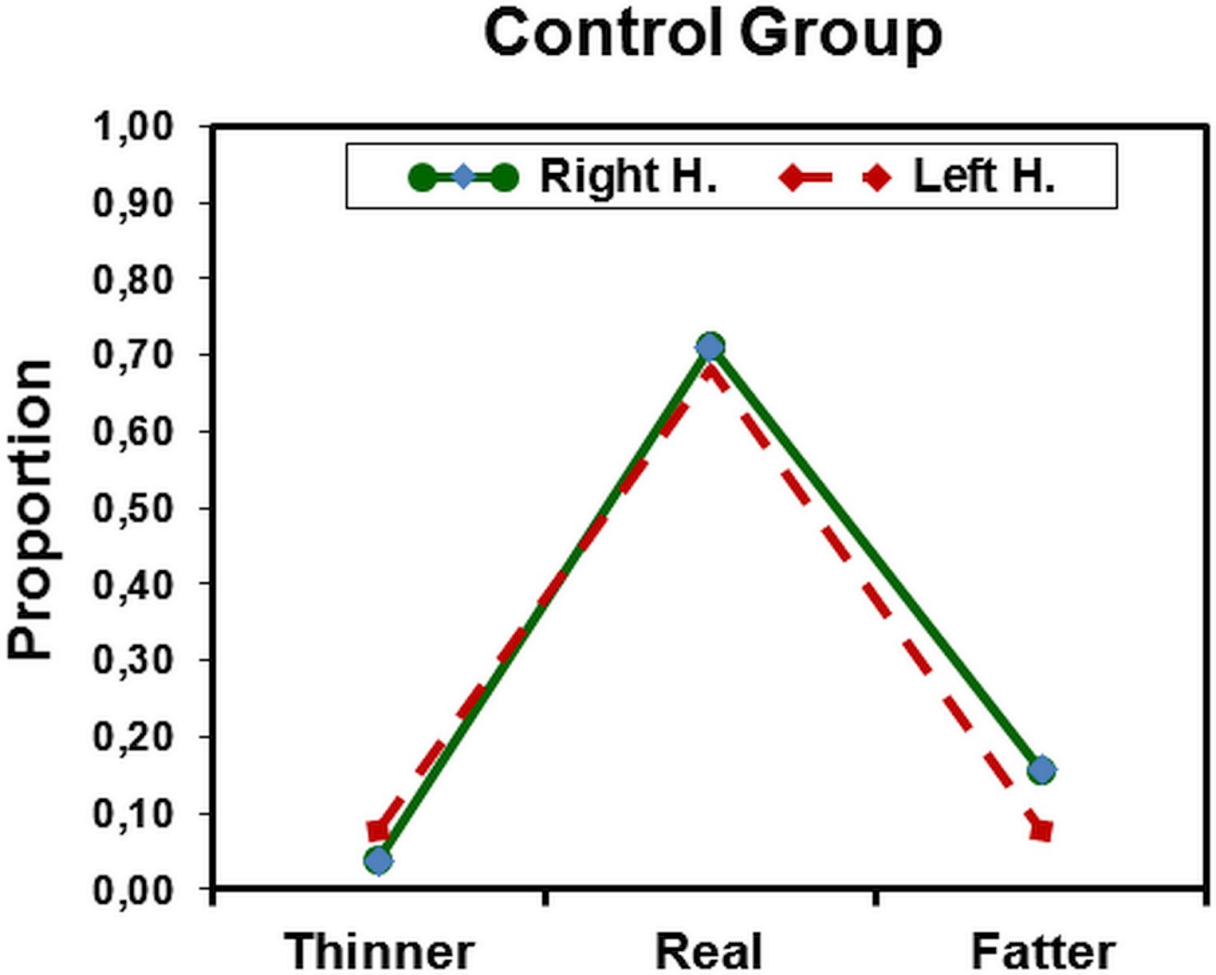
Control healthy group. Mean proportions of equal-to-own-size judgments as a function of body picture distortion for the left (LH) and right hemisphere (RH), respectively, after negative self-related primes. There was no difference between the left- and right hemispheres in any condition.

To test our *a-priori hypothesis* we looked at the ANOVA with *Negative Self primes*. There was a significant main effect of Body Size, and also significant interactions Group x Body Size and Body Size X Visual Field. Most importantly, there was a highly-significant three-way interaction of Group x Body Size x Visual Field: *F*(2, 80) = 47,2, *p* < .00001, partial *η*^2^ = .51. The interaction is depicted in Figs 2 and 3. The most impressive result is that anorexia nervosa patients made a large number of errors when pictures of their own body were presented to the left hemisphere (RVF) but not when they were presented to the right hemisphere (LVF). That is, when they saw the picture of their actual body size they systematically and mistakenly considered it as being fatter than it was (see Fig 2). This pattern was not found for participants in the control group (Fig 3), neither in this prime condition nor with any of the other primes (see Table 2).

As second dependent variable we wanted to use reaction times (cf. Smeets & Kosslyn, [4]). Several participants of both groups, however, did not select any of the thinner (-20%) or fatter (+20%) distortions as equal to their body size, which left corresponding cells for response times empty. Because of that, we analyzed only responses to pictures without body size distortions (Real). The critical condition was *Negative Self Primes*. We therefore carried out a Group (anorexic patients, controls) X Visual Field (left, right) ANOVA on those reaction times. The only significant result was a main effect of Group: *F*(1, 40) = 10.3, *p* < .003, partial *η*^2^ = .20. The anorexic patients had longer reaction times than controls (*M* = 912 ms vs. *M* = 743 ms, respectively). Visual Field or Group X Visual Field were not significant (*F*s < 1).

### "Thinner-than-self" responses

We analyzed accuracy to *Thinner-than-Self* responses using a mixed-design ANOVA including Group (anorexic, controls), Prime (Positive No-Self, Positive Self, Neutral, Negative No-Self, and Negative Self), Body Size (thinner, real, fatter), and Visual Field (left, right), the last three as within-participant factors. There were no differences between anorexic and control participants (all *F*s < 1.0). Prime was not significant either. Results showed an effect of Body Size: *F*(2, 80) = 735.9, *p* < .00001, partial *η*^2^ = .94. Not surprisingly, the proportion of correct responses to pictures thinner-than-self (T) were much more accurate than the incorrect responses to real self (R) or fatter-than (F) own’s body (*M*_T_ = .90, *M*_R_ = .17, *M*_F_ = .02). Visual Field was also significant: *F*(1, 40) = 11.3, *p* < .01, partial *η*^2^ = .22. Accuracy was *higher* for LVF pictures (right hemisphere) than for RVF pictures (right hemisphere): .38 vs. .35, respectively. Finally, there was an interaction between Body Size and Visual Field: *F*(2, 80) = 4.03, *p* < .025, partial *η*^2^ = .09. Accuracy for thinner-than-self responses for pictures presented to the RVF (left hemisphere) was *lower* than for those presented to the LVF (right hemisphere): .87 vs. .93, respectively (*p* < .001). There were no differences between the hemispheres for real size or fatter-than-self responses.

Concerning reaction times of thinner-than (-20%) responses, several participants of both groups did not select any of the real (0%) or fatter (+20%) than self responses. Because of that we carried out an ANOVA on the thinner-than self reaction times with the factors Group (anorexic patients, controls), Prime (Positive No-Self, Positive Self, Neutral, Negative No-Self, and Negative Self), and Visual Field (left, right). We found main effects of Group: *F*(1, 40) = 20.3, *p* < .0001, partial *η*^2^ = .33, and Visual Field: *F*(1, 40) = 55.4, *p* < .0001, partial *η*^2^ = .58. Group x Visual Field was significant: *F*(1, 40) = 8.1, *p* < .007, partial *η*^2^ = .17, as well as Group x Prime x Visual Field: *F*(4, 160) = 3.1, *p* < .025, partial *η*^2^ = .07. The means of the Group x Visual Field interaction were: Patients, LVF (747 ms), Patients, RVF (833 ms), Controls, LVF (638 ms), and Controls RVF (676 ms). That is, patients took longer to respond to thinner-than-self pictures presented on the RVF (left hemisphere).

### "Fatter-than-self" responses

We analyzed accuracy to *Fatter-than-Self* responses with an analogous mixed-design ANOVA as with Thinner-than Self responses. There were no significant differences between anorexic and control participants. Prime was not significant either. There was an effect of Body Size *F*(2, 80) = 505.5, *p* < .00001, partial *η*^2^ = .93. The proportion of correct responses to pictures fatter-than-self (F) were much more accurate than the incorrect responses to real self (R) or thinner than (T) own’s body (*M*_T_ = .01, *M*_R_ = .10, *M*_F_ = .82). Visual Field was also significant: *F*(1, 40) = 7.7, *p* < .008, partial *η*^2^ = .16. In contrast with thinner-than-self responses, accuracy was *lower* for LVF pictures (right hemisphere) than for RVF pictures (left hemisphere): 29.6% vs. 32.4%, respectively.

Concerning reaction times of fatter-than-self (+20%) responses, several participants of both groups did not select any of the real (0%) or thinner-than-self (-20%) responses. Because of that we carried out an ANOVA on the fatter-than-self reaction times with the factors Group (anorexic patients, controls), Prime (Positive No-Self, Positive Self, Neutral, Negative No-Self, and Negative Self), and Visual Field (left, right). The only significant result was the main effect of Group: *F*(1, 35) = 9.4, *p* < .004, partial *η*^2^ = .21. In average, patients were slower (798 ms) than controls (675 ms).

### Correlations between performance data and self report

We examined whether the tendency to make false “fatter-than” body size judgments by anorexic patients when the body pictures were presented to the left hemisphere was related to trait measures. We conducted these analyses by first calculating a *Lateralization Index* based on the frequency of “equal to self” errors made: LH (RVF) fatter—RH (LVF) fatter in the initial part of the experiment. *Positive scores* indicate a tendency to make erroneous judgments of real body pictures presented to the LH (RVF), whereas *negative scores* indicated the tendency to make erroneous judgementswhen they were presented to the RH (LVF). We correlated this index with different questionnaire measures (VCI, ACS & TCQ-E) and separately for each prime condition, including all participants (anorexic patients and controls) to increase power. Results are shown in Table 3. Because there were no significant correlations with the Passive coping scale of the TCQ-E, they are not included. The critical negative self-related prime condition showed many significant correlations, and they were positive (LH bias) for scales related to negative affect and to eating behavior. Negative correlations (RH bias) were found for scales associated with good emotion regulation, flexibility, and self-activation. For the neutral prime condition, on the other hand, there seemed to be a LH bias for some scales related to self-access.

**Table 3:** Correlations between body-image lateralization index and personality measures.

	Positive No-Self	Positive Self	Negative No-Self	Negative Self	Neutral
Self-Determination	.02	-.05	.05	-.45**	.34*
Self-Access	.08	.12	.03	-.50***	.32*
Self-Motivation	.12	.10	.04	-.37*	.37*
Action Orientation Failure	.03	.00	.00	-.36*	.12
Action Orientation Decision	.07	.04	.03	-.32*	.39*
TCQ- Positive Emotions	.00	-.12	-.01	-.30	.07
TCQ-E Low Imp. Control	.01	-.08	-.03	-.45**	-.02
TCQ-Negative Emotions	.15	-.04	.05	.47**	-.22
TCQ-E Distraction	-.04	-.11	.00	.60***	-.20
TCQ-E Negative Emotions	.13	.03	-.21	.64***	-.27
Demands	-.06	.02	-.06	.36*	-.28

Correlations between body-image *lateralization index*: Proportion of “fatter than” errors with real body pictures, LH (RVF)—RH (LVF) and personality measures for each prime condition. Positive correlations indicate a LH bias whereas negative correlations a RH bias in body-image representations.

*Notes*. Data from all participants (anorexia nervosa patients and controls, *n* = 40, two participants missing).

*p < .05,

**p < .01,

*** *p* < .001 (2-tailed)

TCQ = Thought Control Questionnaire; TCQ-E = Thought Control Questionnaire-Eating

### Subsequent activation of the right and left hemispheres

We analyzed accuracy data of body-size judgments after having participants activate their right (left-hand contractions) or left hemisphere (right-hand contractions). We were interested in data of *anorexic patients* with negative self-related primes. For *right hemispheric* (left-hand) activation the Body Size (thinner, real, fatter) X Visual Field (left, right) ANOVA yielded only a main effect of Body Size: *F*(2, 38) = 48.13, *p* = .0001, partial *η*^2^ = .72. For *left hemispheric* (right-hand) activation the ANOVA yielded also only a main effect of Body Size: *F*(2, 38) = 52.52, *p* = .0001, partial *η*^2^ = .33. Neither Visual Field nor the 2-way interaction were significant in either ANOVA.

We also analyzed accuracy data of body-size judgments after having participants of the *control group* activate their right (left-hand contractions) or left hemisphere (right-hand contractions). To compare across groups, we focused on data with negative self-related primes, which was critical for anorexic patients. For *right hemispheric* (left-hand) activation the Body Size (thinner, real, fatter) X Visual Field (left, right) ANOVA yielded only a main effect of Body Size: *F*(2, 42) = 72.01, *p* = .0001, partial *η*^2^ = .77. For *left hemispheric* (right-hand) activation the ANOVA yielded also only a main effect of Body Size: *F*(2, 42) = 82.68, *p* = .0001, partial *η*^2^ = .80. Neither Visual Field nor the 2-way interaction were significant in either ANOVA.

Results are shown in Table 4. There one can see “equal to own size” responses to actual body pictures made by anorexic and control participants for negative self-related primes as a function of hemispheric activation. The main result is that for anorexic patients, responses with “thinner than” and “fatter than” differed significantly for the LH activation condition, with a bias for fatter responses. On the other hand, after RH activation thinner and fatter responses *did not differ* significantly anymore for anorexic patients, indicating a reduction in own body-size distortions. Moreover, after RH activation the anorexic patients showed a similar response pattern as controls after RH activation (see Table 4).

**Table 4:** “Equal-to-own-size” responses made by anorexia nervosa patients and control healthy participants after left- and right-hand activation.

	Thinner (False)	REAL	Fatter (False)
*Anorexia Nervosa Group*			
LH (right hand)	.**10**_**b**_ (.*12*)	.73 (.*17*)	.**24**_**b**_ (.*25*)
RH (left hand)	.08 (.*13*)	.73 (.*22*)	.18 (.23)
*Control Group*			
LH (right hand)	.13 (.*19*)	.80 (.*20*)	.19 (.*21*)
RH (left hand)	.08 (.*16*)	.80 (.*22*)	.16 (.*20*)

Mean proportions (*sd* in parentheses) of “equal-to-own-size” responses to actual body size pictures (0% distortion) made by anorexia nervosa and control healthy participants for *negative self-related primes* as a function of Left- and Right-Hand Activation Condition.

*Note*: Responses to actual body picture as REAL differ significantly from those classified as thinner or fatter than the real body (*p* < .001). For anorexic patients thinner versus fatter responses differed after LH activation (Subscript **b**: *p* < .05), however, they *did not differ* significantly after RH activation. Thinner versus fatter responses did not differ significantly in either activation condition of the control group.

## Discussion

Replicating findings of Smeets and Kosslyn [4] we found that anorexia nervosa patients have a distorted perception of their own body when pictures of their bodies were presented to the left hemisphere (RVF). In contrast, such distortions did not occur when those pictures were prensented to the right hemisphere (LVF). Moreover, this effect occurred only when those pictures were preceded by idiosyncratic negative self-relevant primes. The results are impressive and show a clear-cut pattern, which is depicted in Fig 2. After being primed with negative self-relevant words the patients were unable to accurately estimate their own body size and systematically classified their bodies as "being too fat." As in the study of Smeets and Kosslyn we found that healthy control participants did not have such distorted body representations (see Fig 3). Concerning patients, a review of studies from 2003 to 2013 indicates that body size overestimation is a fundamental feature of anorexia nervosa. The inconsistency of this finding in studies [50] can be attributed to the wide variety of assessment techniques used and their poor psychometric properties. More related to our main hypothesis, there is evidence of the involvement of the left hemisphere in body image distortions (cf. [16]). For example, using fMRI Miyake et al. found that the most negative word related to the body image compared to the most neutral word activated by anorexic-bulimic patients not only the right amygdala [51] but also the *left* medial prefrontal cortex [26]. Using single photon emission computed tomography, Beato-Fernández et al. found abnormalities in the body image of anorexic patients with the Silhoutte test and suggested that they may be related to the storage of a distorted prototypical body image in the *left* parietal lobe [52].

The left-hemispheric bias in body distortions that we observed after negative self-relevant primes confirms PSI theory’s assumption and related findings ([15], [39], [40]) suggesting that these patients function under a self-control executive mode, which maintains unrealistic images ("introjects") of one’s own body size ([16], [52]). Negative beliefs related to the body should be relevant for all persons worried about their own body image but be especially critical for anorexic patients. On this regard, it was recently found that the severity of anorexia nervosa symptoms was related to negative interpretation biases concerning the body, not only for anorexic patients but also for control participants [53].

Beyond cognitive biases, the body distortions of anorexic patients [54] and overweight individuals [55] may be related to a deficit in interoceptive perception, that is a decreased ability to recognize certain visceral sensations related to hunger. There is evidence that normal interoceptive perception is related to activation of the right hemisphere ([19], [20]). Also consistent with PSI theory is the notion that the body distortions did *not* occur when the pictures were presented to the right hemisphere, because its activation should be related to a veridical body representation stored in extension memory ([9], [11]).

As a further step to validate our main finding, that is, the tendency to perceive the body as being too fat when the body pictures were presented to the LH (and with negative self-related primes) we calculated a *Lateralization Index* (LI) based on the frequency of “equal to self” errors made: LH (RVF) fatter—RH (LVF) fatter in the different prime conditions. We then correlated this index with self-report measures. We reasoned that women preoccupied with their own body image, both anorexic patients and healthy controls, would tend to show a LH bias when presented with negative self-related primes. We assumed this because this hemisphere is associated with social norms related to the ideal body image expected by society and to externally induced norms or goals ([8], [9]). Based on the theory of and evidence for “self-infiltration” ([5], [6], [56]) we expected the left hemispheric bias to be observed under negative affect in personally relevant situations. Specifically, we expected that the LI should correlate with scales measuring negative affect and with those related to eating behavior, which could arouse feelings of guilt. Consistent with this expectation, the results in Table 3 for negative self-related primes show that the LI correlates positively (LH bias) with life demands, thought control failure related to negative emotions as well as with negative emotions and distraction related to eating.

On the other hand, given that the RH processes interoceptive information about our own body in a realistic way ([19], [20]), and that the RH is involved in affect regulation and self-access ([9], [11], [13]) we can expect that the LI would correlate negatively with scales assessing successful affect regulation and a positive attitude toward need satisfaction and self-access. Consistent with this expectation, the results listed in Table 3 for negative self-related primes show that the LI correlates negatively (RH bias) with the TCQ-E scales positive emotions, and low impulse control (indicating flexibility or indulgence), with self-management scales (VCI), and with affect regulation scales (AOF and AOD).

The analyses of "thinner-than-self" and "fatter-than-self" responses showed no effects of group or prime condition. There was, however, better performance (higher accuracy and faster reaction times) for thinner-than-self responses for pictures presented to the right hemisphere (LVF). On the other hand, there was better performance (higher accuracy) for fatter-than-self responses for pictures shown to the left hemisphere (RVF). The meaning of these findings is not clear, but they suggest that distorted "fat" body images of oneself are processed more easily if they are presented to the left hemisphere.

In addition, we explored whether the body distortions showed by anorexic patients could be reduced. To accomplish this, we had participants activate their right and left hemispheres through unilateral muscle contractions of the opposite hand. Results showed that RH activation lead to a reduction in the patients’ tendency to perceive their body as being too fat. We found no significant differences between groups regarding the tendency of participants to perceive their body as being too thin. Moreover, under RH activation the pattern of responses in the anorexic patients was similar to that of the healthy participants under no hemispheric activation (compare Tables 2 and 4). On the other hand, LH activation did not help reduce the tendency of patients to perceive the body as being too fat, and this distorted perception was significantly higher than the perception of their body as being too thin (see Table 4). On the other hand, the pattern of responses of healthy participants was not significantly influenced by hemispheric activation, compared to baseline (see Tables 2 and 4).

### Implications for therapy

The aim of cognitive behavioral therapy (CBT) is to change disturbing and unrealistic thoughts related to the body, food, and weight. In an essay on the treatment of eating disorders, Vanderlinden [57] reports that CBT is successful in only about 50% of the cases. He proposes some reasons for the relative success of CBT that include an overemphasis on the content of unrealistic cognitions rather than on patients' inability to stop those cognitions (rumination) in therapy. In addition, the role that family and peers play in supporting problematic behaviors and genetic differences in thinking rigidity might be underestimated, according to Vanderlinden. This author suggests that CBT for eating disorders should be complemented with therapeutic methods such as meditation, or "eye movement desensitisation and reprocessing" (EMDR) therapy, which has been applied successfully in the treatment of post-traumatic stress disorders [58]. In EMDR the patient is requested to generate a vivid visual image related to the critical (traumatic) memory, or a negative belief about the self, related emotions, and body sensations. While doing it the patient is asked to move his/her eyes back and forth following the therapist’s fingers as they move horizontally across the field of vision for about 30 seconds.

Our present findings suggest that the treatment of anorexic patients would also benefit from EMDR therapy. Anorexic patients have a tendency to overactivate the left hemisphere and because those with strong right handedness (LH) show a larger discrepancy between perceived and actual weight (cf. [16]). Therefore, activation of the right hemisphere through left-hand muscle contractions should help anorexic patients restore the balance in activation between the two hemispheres and produce realistic representations of own's body size (as we found in this study). Note that this presumably also be accomplished with EMDR therapy, especially if the patient is presented with *negative statements about the self* during treatment. Findings on the use of EMDR to treat anorexia nervosa are scarce in the literature, but there are some encouraging recent results. Zaccagnino, Cussino, Callerame, Civilotti, and Fernandez [59] report the case of a 17-year-old inpatient girl diagnosed with anorexia nervosa, weighing 28 kg., with a BMI of 14. EMDR therapy was administered for 6 months in hospital, in twice weekly 50-min sessions. These sessions consisted of standard procedures primarily focusing on her relational traumas as well as talk therapy sessions, which were integrated with "ego-state" therapy. The results were positive and at the end of the therapy she weighted 55 kg., with a BMI of 21.5. She also no longer fulfilled the criteria for diagnosis of anorexia nervosa. Another recent paper from the same authors contrasts CBT with EMDR therapy and provides guidelines for the treatment of anorexic patients [60].

## Conclusions

We show that anorexia nervosa patients have a left-hemispheric bias in body distortions after being reminded of their negative self-aspects. This tendency is ameliorated when participants activate their right hemisphere (left-hand muscle contractions). The results therefore suggest two ways of treating anorexic patients: (a) Increasing their sense of self-worth and self-esteem, and (b) activating their right hemisphere. Therapy approaches based on CBT should be complemented with alternative methods, such as left-hand muscle contractions or EMDR. More generally, the main focus of therapy should be placed on trying to change anorexic patients’ preferred mode of functioning from self-control to self-regulation, that is from the strict (self-denying) to the soft (self-indulgent) form of self-management [9]. There are different ways of achieving this goal and include aspects such as clarifying whether the goal of “being thin” is really self-compatible or introjected [6], increasing autonomy support, moving from external to internal levels of motivation [10], or increasing their authentic self-motivation in goal pursuit ([13], [15]).

## Supporting information

S1 Data Spreadsheet with proportions of responses of the anorexia nervosa patients and control pariticipants under various prime- and body-distortion conditions. BMI, handedness score, and age of participants as well as their scores in the personality tests reported in the results' section are also included.
(XLSX)
